# The Bioengineering of Microspheric Skin Organoids and Their Application in Drug Screening

**DOI:** 10.1002/advs.202416863

**Published:** 2025-05-08

**Authors:** Jundong Xie, Qingyang Yang, Yanan Zhang, Ke Zheng, Hongya Geng, Yaojiong Wu

**Affiliations:** ^1^ State Key Laboratory of Chemical Oncogenomics and Institute of Biopharmaceutical and Health Engineering (iBHE) Tsinghua Shenzhen International Graduate School Tsinghua University Shenzhen 518055 China; ^2^ Key Laboratory of Active Proteins and Peptides Green Biomanufacturing of Guangdong Higher Education Institutes Tsinghua Shenzhen International Graduate School Tsinghua Shenzhen International Graduate School Tsinghua University Shenzhen 518055 China

**Keywords:** bilayered skin model, drug screening, in vitro skin model, microspheric skin, skin organoid

## Abstract

It has been a challenge to develop skin models which exhibit the key characteristics of the human skin and facilitate high‐throughput drug screening. In this study, a method is developed to construct microspheric skin organoids based on spinning bioreactors. The organoid consists of a core‐shell structure to mimic the bilayered skin structure, where the shell is composed of cultured human keratinocytes, resembling the epidermis, and the core, which mimics the dermis, comprises human dermal fibroblasts and collagen. In fabrication of the organoid, the cores are cultured with keratinocytes in spinner flasks under defined conditions to facilitate epidermal growth and differentiation to form mature barrier. To enable efficient drug screening, the organoid is equipped with a luciferase reporter to detect canonical Wnt activation, and Minoxidil is identified to induce epidermal Wnt/beta‐catenin pathway signaling. Thus, the study has developed a novel method for efficient preparation of uniform microspheric skin organoids with potential applications for high‐throughput drug testing and screening.

## Graphical abstract

A novel method for the preparation of uniform microspheric skin organoids via spinning bioreactors is developed. The organoid consists of a core‐shell structure to mimic the bilayered skin structure with the epidermis on the surface. For efficient drug screening, the organoid is equipped with a luciferase reporter to detect canonical Wnt signaling, and Minoxidil is identified as an activator of the pathway.

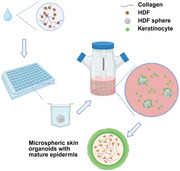

## 1 Introduction

The skin is the largest organ in the body, covering the entire external surface and serving as a protective barrier against external insults. It comprises three layers: the epidermis (outermost), dermis, and hypodermis. The epidermis is primarily made up of stratified keratinocytes, which originate from stem cells in the basal layer and mature as they migrate upward to form the stratum corneum, which is composed of keratin and dead cells.^[^ ^1^, ^2^ ^]^ The dermis contains fibroblasts that secrete growth factors and extracellular matrix (ECM) molecules, nourishing and supporting the epidermis.

The health of the skin depends on the interplay between its cells and ECM components, with bioactive factors playing a crucial role in maintaining homeostasis and promoting regeneration. For example, the epidermal activation of canonical Wnt signaling pathway plays a central role in hair follicle development and regeneration.^[^ ^3^, ^4^ ^]^ It is desirable to develop efficient tools to discover bioactive factors from pools of compounds, peptides, and nucleic acids for skin health and disease treatments.

Skin organoids hold great promise in studying organogenesis, disease modeling, drug discovery, and regenerative medicine.^[^ ^5^, ^6^, ^7^ ^]^ In the past, several skin models have been developed as powerful tools for drug testing and discovery. First, planner epidermal equivalents consisting of stratified keratinocytes are developed, and have been used extensively for cytotoxicity tests of cosmetics.^[^ ^8^, ^9^ ^]^ Then, a bilayered skin sheet containing both the epidermal and dermal layers has been constructed and used for skin permeability tests and many other applications.^[^ ^10^ ^]^ Though these culture‐well‐based planner skin models have been commercialized for cosmetic testing, their applications in drug screening and discovery are restricted due to low efficiency in fabrication and testing.^[^ ^11^, ^12^ ^]^

Multicellular 3D microspheric models have emerged recently as a promising approach for high‐throughput drug screening. One study reported a melanoma cell skin invasion model, where bilayered skin spheroids with HaCaT keratinocytes on the surface were cultured with melanocytes, serving as a model to study cancer cell invasion and evaluate drug effectiveness.^[^ ^13^ ^]^ In a more recent study, a microspheric skin model was constructed by coculturing keratinocytes, fibroblasts, and endothelial cells on non‐adherent tissue culture wells, where cells aggregated and formed an exterior epidermal layer. However, these organoids were prepared based on tissue culture wells, and the methodology normally yields cell aggregates in ununiform size and does not support large scale fabrication. Nevertheless, the studies have opened up a new avenue for spheric skin organoid research.

This study addresses these limitations by developing an efficient method using spinner cultures to fabricate uniform bilayered microspheric skin organoids, where primary human keratinocytes were induced to proliferate and fully differentiate into mature epidermal structures. In addition, a luciferase reporter for canonical Wnt activation was introduced into the keratinocytes to facilitate drug screening, and Minoxidil was identified to induce epidermal Wnt activation, offering new insights for skin therapeutics.

## 2 Results and Discussion

### 2.1 Genesis of Microspheric Skin Organoids

**Figure 1:**
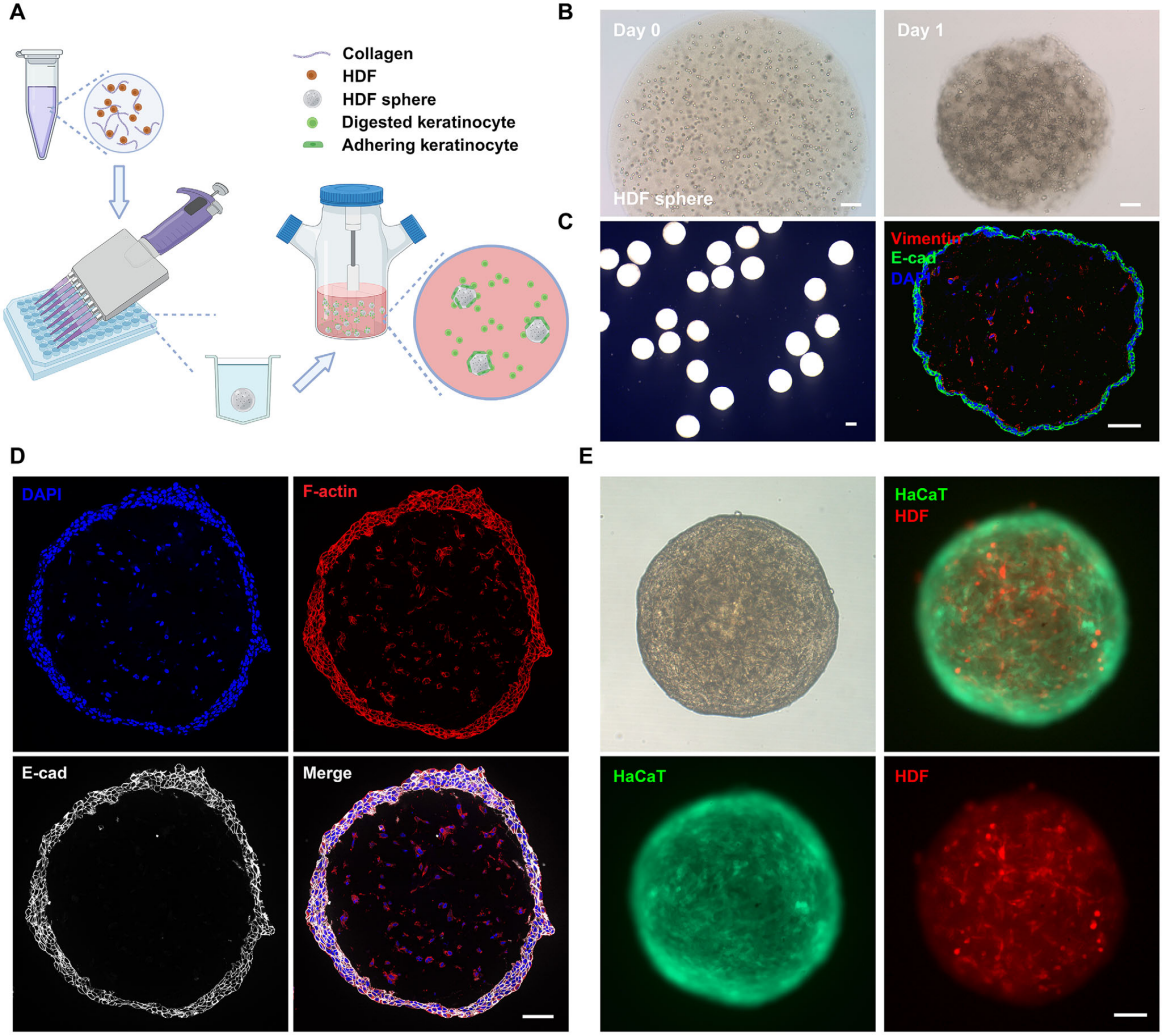
Fabrication of microspheric skin organoids. A). A schematic diagram (Created in BioRender) illustrating the formation of microspheric skin organoids. B). Photographs of human dermal fibroblast (HDF)‐spheres at day 0 and day 1 under a light microscopy. C). Representative images of day 5 microspheric skin organoids (left), and the cross section of the organoid after immunofluorescent staining for E‐cadherin (E‐cad) and vimentin. D). Representative images of cross‐sections of a day 7 organoid after immunofluorescence staining for F‐actin and E‐cad. E). Representative microscopic images of a day 7 microspheric skin organoid (whole mount) showing a core‐shell structure, with the core composed of mCherry‐expressing HDFs (red) and the shell formed by GFP‐expressing HaCaT cells (green). Scale bar: 100 µm.

The fabrication of microspheric skin organoids was achieved by a two‐step method that established a core‐shell structure (**Figure** **1**A). Initially, human dermal fibroblasts (HDFs) were combined with a collagen‐I solution and deposited into non‐adherent wells, forming HDF‐spheres (≈1800 µm in diameter). Within 24 h, the diameter of HDF‐spheres decreased considerably to ≈1000 µm due to HDFs‐mediated hydrogel contraction (Figure 1B). Subsequently, HDF‐spheres were transferred to spinner flasks and co‐cultured with HaCaT cells, which adhered to the surface of HDF‐spheres and developed an epidermis‐like shell. To track cellular dynamics during organoid formation, HDFs were labeled with mCherry, and HaCaT cells were marked by green fluorescent protein (GFP). The in vitro co‐culture of HDF‐spheres (core) and HaCaT cells (shell) resulted in uniformly sized organoids with a distinct core‐shell architecture (Figure 1E). Immunofluorescence analysis of cross‐sections of the organoid revealed a stratified epidermal shell composed of keratinocytes expressing high levels of E‐cadherin, indicative of strong cell‐cell adhesion. Meanwhile, HDFs in the inner core, which were marked by the expression of vimentin and F‐actin, were well spread with processes, suggesting robust attachment to the collagen matrix (Figure 1C,D).

The optimal condition for organoid fabrication was investigated, and several factors were found critical, such as cell number per sphere, the ratio of epidermal and dermal cells, and the stirring speed of the culture. The size of the spheres was negatively related to the number of HDFs, likely due to HDF contraction, and the size of an HDF‐sphere containing 2000–4000 HDFs was 500–800 µm in diameter with a regular shape (Figure S1A–D, Supporting Information). Excess HDFs increased cell death in the core likely due to insufficient nutrition. Additionally, the stirring speed affected the adhesion of keratinocytes to the surface of HDF‐spheres, and a stirring speed of 20 rpm yielded maximal adhesion of the keratinocytes (Figure S1B–E, Supporting Information). Moreover, the ratio of keratinocytes to HDFs critically affected the formation of the epidermal layer, and a ratio of 10:1 ensured the timely development of the epidermal layer without causing inter‐sphere adhesion (Figure S1C, Supporting Information). Hereafter these parameters were used for organoid preparation.

### 2.2 Characteristics of Microspheric Skin Organoids

**Figure 2:**
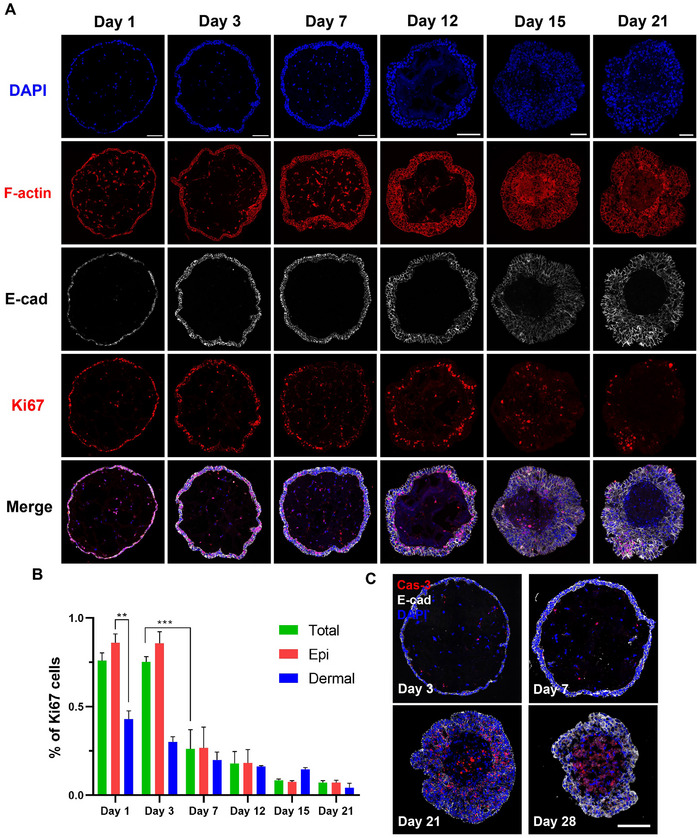
Characteristics of microspheric skin organoids. A). Representative confocal images of organoids cultured for varying durations, where epidermal and dermal cells were stained for F‐actin, and keratinocytes were marked by E‐cadherin (E‐cad). B). Quantification of the percentages of Ki67^+^ epidermal (Epi) and dermal cells in the organoids, respectively, which were cultured for different times. All values are presented as mean ± SEM (*n* ≥ 3). Statistical significance: one‐way ANOVA (^**^ *p* < 0.01, ^***^ *p* < 0.001). C). Representative confocal images showing caspase‐3 positive (red) apoptotic cells in the organoids which were cultured for 3, 7, 21, and 28 days, respectively. Scale bars: 100 µm.

To understand the formation process of microspheric skin organoid, we analyzed organoids cultured at different time points. On day 1, a monolayer epidermal structure was formed by HaCaT cells on the surface of the HDF‐spheres, while HDFs in the core displayed a spread morphology with visible F‐actin bundles. As co‐culture progressed, the epidermal layer thickened, forming two layers by day 3 and three layers by day 7. By day 21, a stratified epidermis with up to seven cell layers had developed, though its thickness was uneven. Despite this increase in epidermal layers, the overall volume of the organoids did not expand significantly, primarily due to the shrinkage of the HDF core caused by HDF contraction. Notably, keratinocytes maintained high expression of E‐cadherin and intact cell‐cell junctions throughout epidermal thickening (**Figure** **2**A).

To assess cell growth and viability, we examined the proliferation and apoptosis of the organoid during culture. Ki67 staining indicated high proliferation rates in both HaCaT cells and HDFs during the first three days (Figure 2B). However, the proliferation rate declined sharply thereafter, with only a small fraction of proliferating cells by day 21. The detection of apoptotic cells using cleaved caspase‐3 staining revealed minimal cell death in both the epidermal and dermal compartments in the first week. However, cell apoptosis increased significantly in late stages (days 21 and 28), particularly in the basal layer of the epidermis and the HDF core, likely due to nutritional insufficiency (Figure 2C). Our findings suggested that microspheric skin organoids exhibited distinct characteristics at different stages, which may be leveraged for various applications in research and drug screening.

### 2.3 Microspheric Skin Organoids for Drug Testing

**Figure 3:**
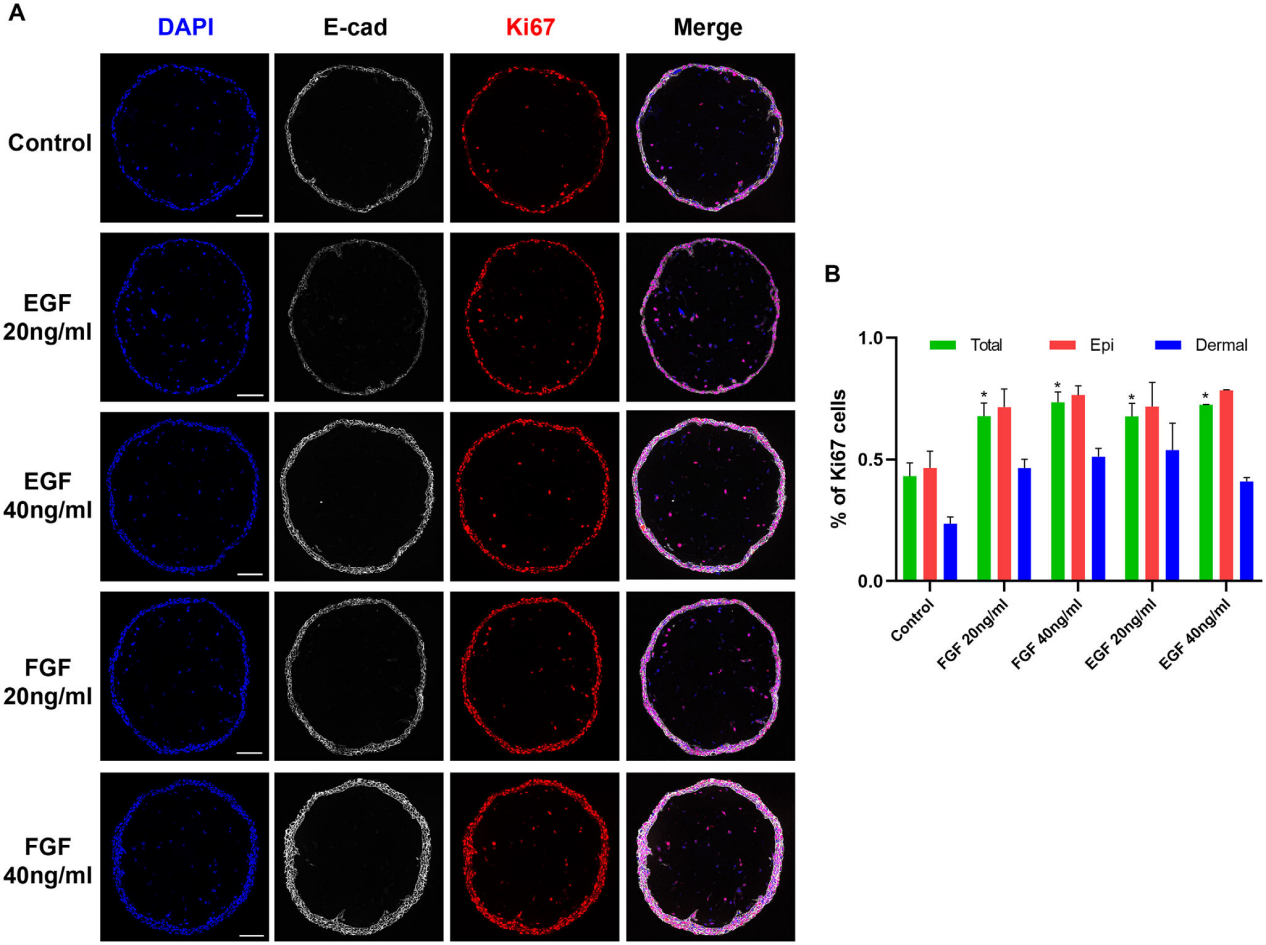
The effect of growth factors on microspheric skin organoids. A). Representative confocal images of the skin organoids treated with vehicle medium (control), 20 ng mL^−1^ EGF, 40 ng mL^−1^ EGF, 20 ng mL^−1^ bFGF, and 40 ng mL^−1^ bFGF, respectively. Keratinocytes were stained positive for E‐cadherin (E‐cad), nuclei were stained blue by DAPI, and proliferating cells were marked by Ki67. B). The percentages of Ki67^+^ proliferating cells in the entire organoid comprising HaCaT cells, the epidermis (Epi), and the dermal core were counted, respectively. All values are presented as mean ± SEM (*n* ≥ 3), ^*^ *p* < 0.05. Scale bars: 100 µm.

To evaluate the potential of microspheric skin organoids for drug testing, we examined the effects of compounds known to influence epidermal growth. Organoids were first cultured for two days, allowing the formation of a two‐layered epidermal structure, and then incubated in non‐adherent culture wells with or without epidermal growth factor (EGF, 20 or 40 ng mL^−1^), or basic fibroblast growth factor (bFGF, 20 or 40 ng mL^−1^) for 48 h. As expected, both EGF and bFGF significantly increased the proportion of Ki67^+^ proliferative cells in both the epidermal and dermal compartments, leading to a thicker epidermis (**Figure** **3**A,B).

To further explore the suitability of organoids comprising primary human keratinocytes for drug testing, organoids were incubated with epidermal growth medium or differentiation medium one day before drug exposure, and then treated with CHIR‐99021 (5 or 10 µm), EGF (20 or 40 ng mL^−1^), or bFGF (20 or 40 ng mL^−1^) for 48 h. All three compounds exhibited dose‐dependent pro‐proliferative effects, regardless of the differentiation stages of organoids. However, organoids with a more mature epidermal barrier were less responsive to drug treatment, particularly to CHIR‐99021 (Figure S3, Supporting Information).

**Figure 4:**
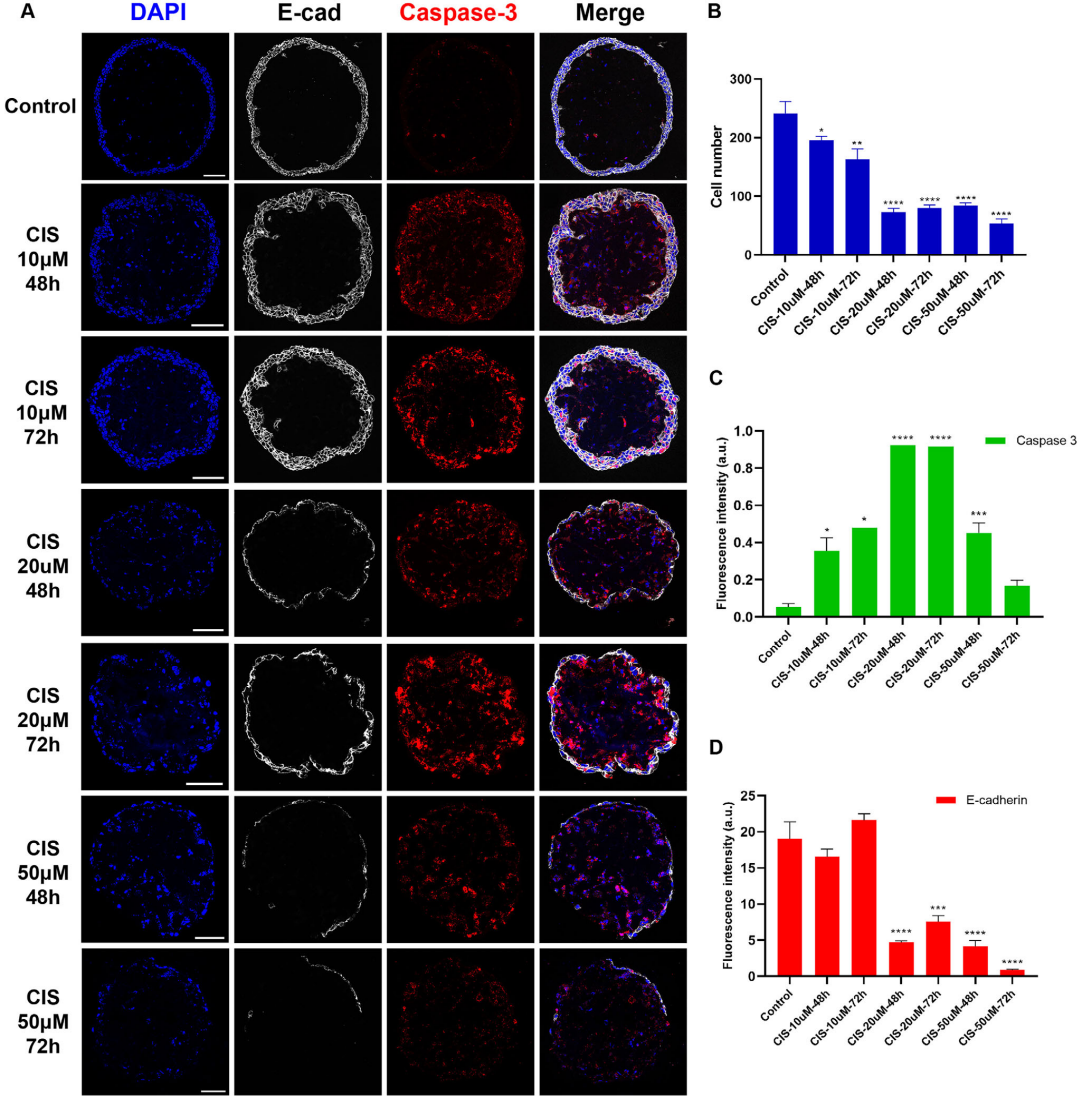
The effect of cisplatin on microspheric skin organoids. A). Representative confocal images of the skin organoids treated with different concentrations of cisplatin (CIS) for different times. Keratinocytes were stained positive for E‐cadherin (E‐cad), nuclei were stained blue by DAPI, and apoptotic cells were detected by cleaved caspase‐3. (B–D). Quantification of cellular changes of the organoids after cisplatin treatment. B). Changes in total cell number. C). Changes in expression levels of E‐cadherin. D). Changes in expression levels of cleaved caspase‐3. All values are presented as mean ± SEM (*n* ≥ 3), ^*^ *p* < 0.05, ^**^ *p* < 0.01, ^***^ *p* < 0.001. Scale bars: 100 µm.

Similarly, organoids were treated with cisplatin, a compound known to block DNA synthesis, at concentrations of 10, 20, and 50 µm. Epidermal cell proliferation and apoptosis were assessed at 48 and 72 h, respectively. The results indicated that cisplatin decreased the number of keratinocytes in a dose‐dependent manner at concentrations of 10 and 20 µm, but no additional reduction was observed at the 50 µm (**Figure** **4**A,B). Conversely, the proportion of apoptotic cells, as indicated by cleaved caspase‐3 staining, increased consistently with higher doses of cisplatin (Figure 4A,C). Further analysis revealed distinct structural changes in epidermal integrity after cisplatin treatment. For organoids treated with 10 µm cisplatin, keratinocytes maintained strong cell–cell adhesion, marked by E‐cadherin levels similar to the control group, despite an increase in cell apoptosis (Figure 4A,D). However, cell–cell junctions in the organoids treated with 20 µm cisplatin were visibly disrupted, indicating a compromised epidermal barrier. These findings highlighted the potential of microspheric skin organoids as a reliable model for assessing drug effects.

### 2.4 Microspheric Skin Organoids with Signaling Reporter for Drug Discovery

**Figure 5:**
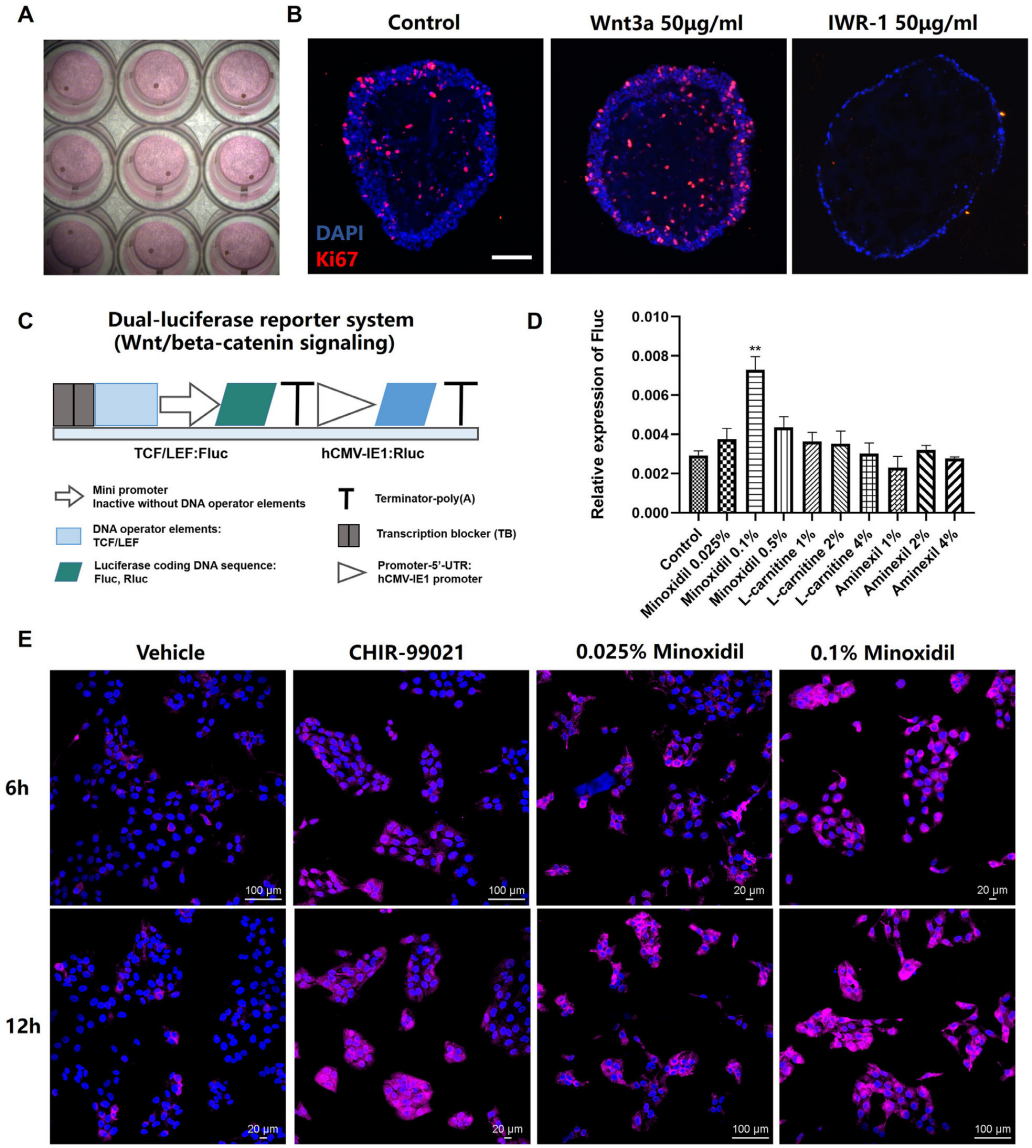
Microspheric skin organoids bearing dual‐luciferase reporter system for assessment of Wnt/beta‐catenin pathway activation. A). One microspheric skin organoid per well was seeded into 96‐well plate. B). The effect of Wnt3a and IWR‐1on cell proliferation in the organoids. Representative confocal images of the organoids treated with vehicle medium (Control), 50 µg mL^−1^ Wnt3a, and 50 µg mL^−1^ IWR‐1 for 48 h. Scale bars: 100 µm. For each condition, ≥3 microspheres were analyzed. C). A diagram illustrating dual‐luciferase reporter system used for detection of Wnt signaling pathway activation. Redrew based on a multiplex hextuple luciferase assay.^[^ ^19^ ^]^ D). Microspheric skin organoids with dual‐luciferase reporter system in screening for compounds to activate the Wnt pathway. Compounds including Minoxidil (0.025%, 0.1%, and 0.5%), L‐carnitine (1%, 2%, and 4%), and Aminexil (1%, 2%, and 4%) were incubated with the organoids, respectively, and D‐luciferin and coelenterazine were sequentially added to detect Rluc and Fluc. The expression levels of Fluc were presented as the value of Fluc relative to Rluc. Values were presented as mean ± SEM (*n* ≥ 3), ^*^ *p* < 0.05, ^**^ *p* < 0.01, ^***^ *p* < 0.001 E). The effect of Minoxidil on the activation of Wnt/beta‐catenin signaling pathway. HaCaT cells were treated with Minoxidil (0.05% and 0.1%) for 6 and 12 h, and cells treated with vehicle medium alone or 10 µm CHIR‐99021 served as negative or positive control. After treatments, the cells were immunofluorescence stained for beta‐catenin (red), and the activation levels of Wnt/beta‐catenin signaling pathway were reflected by the fluorescence intensity.

The Wnt/β‐catenin signaling pathway is essential for hair follicle development and regeneration.^[^ ^14^, ^15^ ^]^ We observed that the addition of Wnt3a (50 µg mL^−1^) into the culture significantly increased the number of Ki67^+^ proliferating keratinocytes in the organoids. In contrast, IWR‐1 (50 µg mL^−1^), a known inhibitor of this pathway, markedly suppressed keratinocyte growth (**Figure** **5**A,B). These findings confirmed that Wnt/β‐catenin signaling influences cell proliferation in the organoids, establishing a foundation for its use in drug discovery.

To enable high‐throughput drug screening for skin regeneration, we incorporated a luciferase‐based reporter system into the organoids to monitor Wnt/β‐catenin activity, which is typically reflected by the activation of TCF/LEF binding sites. TCF/LEF dual‐luciferase reporter system was integrated into the keratinocytes (HaCaT cells) or the fibroblasts (HDFs) via lentiviral delivery, respectively. This system contained two regulatory DNA domains: TCF/LEF and hCMV‐IE1, in which Rluc served as a baseline control and Fluc detected the activation of TCF/LEF domains (Figure 5C). The background activity of Rluc was measured after adding D‐luciferin, while the Wnt‐responsive activity of Fluc increased following a 12 h treatment with Wnt3a (200 ng mL^−1^), validating the sensitivity of this reporter system (Figure S2, Supporting Information).

Using this reporter system in organoids, three compounds (Minoxidil, L‐carnitine, and Aminexil) were evaluated, which have shown effects with varying certainty on hair growth in previous studies,^[^ ^16^, ^17^, ^18^ ^]^ and are suggestive of potential activities on Wnt signaling pathway. Among them, 0.1% Minoxidil significantly elevated the Wnt‐responsive activity of the Fluc, suggesting it activated Wnt signaling pathway (Figure 5D).

To confirm Minoxidil's impact on Wnt/β‐catenin signaling, HaCaT cells or primary human keratinocytes were incubated with Minoxidil at concentrations of 0.025% and 0.1%, and immunofluorescence staining of β‐catenin revealed a dose‐dependent increase in β‐catenin levels in both cell types. Notably, 0.1% Minoxidil in HaCaT cells increased the expression of β‐catenin to a level comparable to those induced by 10 µm CHIR‐99021, a known Wnt pathway activator, with much of the β‐catenin located in the cell nuclei (Figure 5E). These findings demonstrated that organoids equipped with Wnt reporter system can effectively identify compounds like Minoxidil that enhance signaling critical for skin regeneration.

### 2.5 Epidermal Differentiation and Formation of Mature Barrier in the Organoids

**Figure 6:**
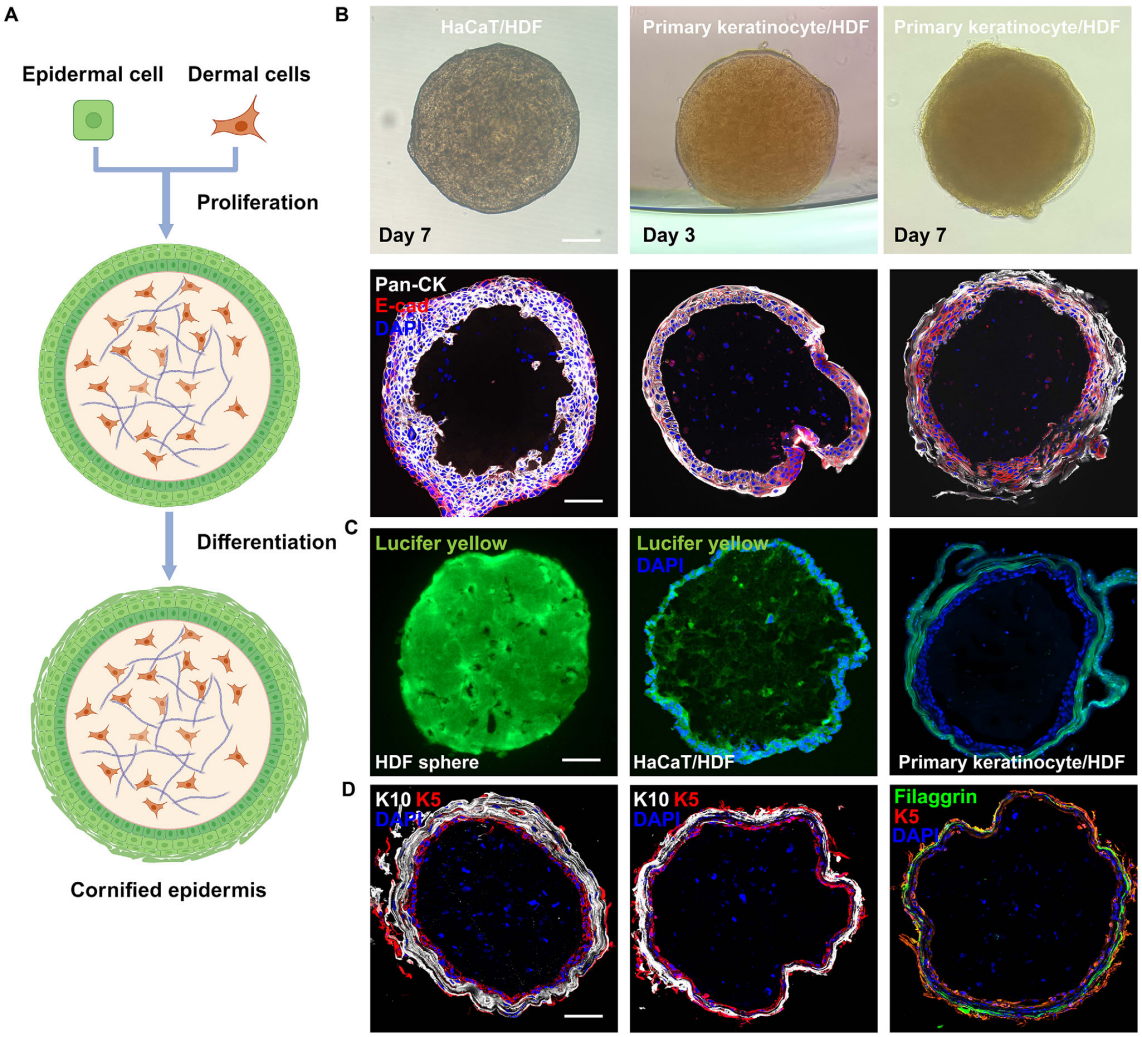
Terminal differentiation of the epidermis in microspheric skin organoids. A). A schematic diagram (Created in BioRender) illustrating the formation of cornified microspheric skin organoids. Organoids consisting of primary human keratinocytes and HDFs were first cultured in growth medium to allow keratincoytes to proliferate, and then in induction medium to induce epidermal maturation. B). Representative images of microspheric skin organoids prepared by different keratinocytes. Cytokeratins were stained using a pan‐cytokeratin (Pan‐CK) antibody, E‐cadherin (E‐cad) was stained in red, and nuclei were stained blue. C). Lucifer Yellow permeability assay of the human dermal fibroblast core (HDF, left), skin organoids with epidermis formed by HaCaT cells (middle), or by primary human keratinocytes (right), where the dye was in green color and the nuclei were in blue. D). Sections of day 7 organoids comprising primary human keratinocytes were stained for keratin 5, keratin 10, and filaggrin to illustrate the differentiation of the epidermal cells. Scale bars: 100 µm.

In human skin, keratinocytes in the epidermis differentiate to form stratified layers, producing cytokeratins that vary across the layers,^[^ ^2^ ^]^ The outermost layer of the epidermis, known as the stratum corneum, forms a critical barrier and represents the mature differentiation of keratinocytes.^[^ ^20^, ^21^ ^]^ To replicate this in the microspheric skin organoids, the organoids were cultured sequentially in growth medium followed by differentiation medium to achieve mature differentiation of the epidermis (**Figure** **6**A). Immunofluorescence co‐staining of cytokeratins (detected broadly across keratinocytes) and E‐cadherin (present in most keratinocytes except for those in the stratum corneum) was performed to assess the structure of the epidermis. Multiple epidermal layers formed in the organoids with HaCaT cells, expressing both E‐cadherin and cytokeratins, but the stratum corneum did not develop even after induction for differentiation (Figure 6B).

To achieve full epidermal differentiation and the formation of mature barrier, we constructed organoids using culture‐expanded primary human keratinocytes (epidermal stem cells, Epi‐SCs) and HDFs. When cultured in growth medium, primary keratinocytes rapidly adhered to the surface of HDF‐core and proliferated, forming a stratified epidermis‐like structure, where the keratinocytes expressed both pan‐cytokeratin and E‐cadherin, similar to HaCaT cells (Figure 6B). By day 3, the outermost epidermal cells began to flatten, suggestive of the onset of differentiation.

Upon switching to differentiation medium, the outermost epidermis of organoids became progressively rough and irregular. By day 7, immunofluorescence staining showed that the expression of E‐cadherin was restricted to the inner epidermal layers, while pan‐cytokeratin stained all the epidermal cells. However, the nuclei of cells in the outmost epidermal layer were absent. These structural changes indicated the formation of the stratum corneum (Figure 6B). The barrier function of the organoids was then determined by Lucifer Yellow permeability assay. Lucifer Yellow is a fluorescent dye that cannot be absorbed by live cells or transported across cell membranes, which makes it ideal for assessing cell junctions and the barrier integrity of the epidermal layer. The results showed that only the outermost stratum corneum was stained with the dye in the organoids comprising primary keratinocytes, whereas the dye stained the entire epidermal layer and the dermal core in the organoids consisting of HaCaT cells (Figure 6C). Filaggrin is a structural protein that is fundamental in the development and maintenance of the skin barrier.^[^ ^22^ ^]^ Immunofluorescence staining indicated that keratin 5 (K5) was expressed in the basal epidermal layer, K10 was expressed in the suprabasal layers (spinous and granular layers), and filaggrin was located in the stratum granulosum, similar to the structure of the normal human skin (Figure 6D). These findings indicated the mature barrier function of the skin organoids, establishing their value as reliable in vitro skin models for evaluating skin barrier function.

## 3 Discussion

Microspheric skin organoids have the potential to facilitate high throughput drug screening, thus overcoming the limitations with planner skin organoids. In this study, we developed a bioreactor‐based method to prepare microspheric skin organoids in uniform size with scalability. The organoids consisted of two compartments, the exterior epidermis‐like structure formed by stratified keratinocytes, and the interior dermis, composed of dermal fibroblasts and collagen. The structure simulates the epidermis and dermis of the human skin, with the epidermis exposed to the outside environment. The organoids are structurally different from the skin spheroids reported in a recent study, whose fibroblasts are on the outside of keratinocytes.^[^ ^23^ ^]^ Recently, reports have indicated that microfluidic technology, which offers a novel method for constructing core‐shell structures with an epidermis layer on the outside, has been utilized to create skin microspheres applicable for skin transplantation and hair neogenesis.^[^ ^24^, ^25^ ^]^ Additionally, extracellular matrix‐free spheric skin organoids with exterior epidermal layers have been reported in two relevant studies, which were formed solely by self‐assembly of skin cells in non‐adherent wells.^[^ ^13^, ^26^ ^]^ With inherited limitations of this method, organoids often vary considerably in size and structure, and are technically unsuitable for large‐scale fabrication. To address these limitations, we developed a two‐step method for the preparation of spheric skin organoid, first preparing uniform spheric dermal cores in defined amounts of fibroblasts and collagen hydrogel, and then coculturing the dermal cores with keratinocytes in spinning flasks in defined conditions. This method generated spheric organoids in uniform size and structure.

Our microspheric skin organoids have displayed several properties to satisfy the need for translational applications. They can be fabricated in large‐scale while maintaining high reproducibility and uniformity throughout the manufacturing process. Besides, it is cost‐effective and amenable to automation. Moreover, the method can be adapted for other heterocellular models including tumor organoids and the organoids mimicking specific organs by incorporating specific cellular components, thus allowing for the creation of personalized organoids.

We found that primary keratinocytes in the spheric organoids could fully differentiate to form mature epidermis upon appropriate induction. Air‐liquid interface culture has been used to promote the formation of the stratum corneum, a structure reflecting mature differentiation of the epidermis, in the preparation of planar epidermal equivalents.^[^ ^20^, ^21^, ^27^, ^28^ ^]^ However, this method is unsuitable for bioreactor culture. In this study, we found that sequential culture of spheric organoids in growth medium followed by induction medium (containing a high concentration of calcium) in spinning flasks promoted the proliferation and subsequent differentiation of primary human keratinocytes adhered to dermal cores, resulting in stratified epidermis with a stratum corneum layer, which was similar to the structure of native human epidermis. The mechanisms underlying the mature differentiation of the keratinocytes in our stirring bioreactor‐based culture are not fully understood. High concentration of calcium in the culture medium probably plays an important role. It is well established that calcium serves as the major mediator of keratinocyte differentiation in vivo and in vitro, and that high concentrations of calcium promote keratinocyte differentiation and maturation.^[^ ^29^, ^30^ ^]^ In addition, mechanical forces such as shear stress and stretch in string culture may promote keratinocyte differentiation. As the primary cells covering the surface of our body, keratinocytes constantly monitor and respond to changes in their environment. Mechanotransduction is known to influence the proliferation and differentiation of many cell types.^[^ ^31^, ^32^ ^]^ Specifically, it induces YAP activation in keratinocytes, which plays important roles in cutaneous wound healing and regeneration in mice.^[^ ^33^, ^34^ ^]^

HaCaT cells are a spontaneously immortalized human keratinocyte line that has been widely used for studies of skin biology and differentiation. HaCaT cells have an infinite lifespan and can be passaged many times without senescence, while primary keratinocytes have a limited lifespan and undergo senescence after a finite number of passages. We found that HaCaT cells formed a multilayered epidermis‐like structure in the organoid with tight intercullular adhesion, but did not fully differentiate into the stratum corneum after induction, which were different from primary human keratinocytes. These results indicate that HaCaT cells have a more limited differentiation potential compared to primary keratinocytes. This finding is consistent with previous studies.^[^ ^35^, ^36^ ^]^

In this study, we introduced a luciferase reporter for canonical Wnt activation into both epidermal and dermal cells of the organoid, and validated its effectiveness in monitoring the activity of the signaling pathway. Notably, with the organoid we identified Minoxidil as a compound capable of inducing epidermal Wnt/beta‐catenin pathway activation from several candidates. The activation of Wnt/beta‐catenin signaling pathway in epidermal cells has been known to be crucial for hair follicle development and regeneration.^[^ ^14^, ^15^ ^]^ Minoxidil is a medicine primarily used to treat high blood pressure by promoting vasodilation. Additionally, it has been observed to stimulate hair growth, but the underlying mechanisms are yet to be fully elucidated. In a previous study, topical application of Minoxidil on the skin of mice induced prolonged hair follicle anagen associated with activation of beta‐catenin pathway in dermal papilla cells.^[^ ^37^ ^]^ We validated the effect of Minoxidil on keratinocytes, and found that treatment of HaCaT cells and primary human keratinocytes with Minoxidil increased the level of beta‐catenin and its translocation to the nuclei, indicating enhanced activation of the signaling pathway. The results suggest a novel mechanism by which Minoxidil promotes hair regeneration.

Despite that the microspheric skin organoid generated in this study has simulated the basic structure of the human skin, it lacks some important components and structures, such as melanocytes, immune cells, blood vessels, and appendages. It is feasible to add some of these cells to the spheric organoids to meet specific testing purposes. For example, to incorporate melanocytes into the epidermal compartment to form an organoid for pigmentation testing, and to add immune cells to form an organoid for inflammation analysis. However, this may involve corresponding optimizations of the coculture conditions. As an alternative approach, several recent studies have used pluripotent stem cells to generate skin organoids with appendages in vitro,^[^ ^38^, ^39^ ^]^ which provide a powerful tool to study skin development and disease mechanisms. In this study, we aimed to develop a spheric skin organoid that facilitates high throughput drug testing and discovery, with a priority on the reproducibility, cost‐effectivity, and scalability of the model.

Automatic high‐throughput drug screening based on this skin organoid can be achieved by integrated application of current commercial platforms. For example, the Cellario scheduling software can be utilized for automated process control, while the spheroONE can be adjusted for organoid sorting in batch.^[^ ^40^, ^41^ ^]^ Besides, the integration of non‐contact liquid handling system, automated incubators, and microplate readers also facilitates drug screening, from high‐throughput drug supplementation to organoids culture, and endpoint analysis. However, successful integration of the organoids into standardized screening workflows still faces challenges, including establishing organoid sensitivity thresholds to common drug solvents, optimizing initial drug concentrations, and validating appropriate statistical methods. Therefore, systematic optimization is necessary prior to applications.

## 4 Conclusion

We have developed a method to create uniform microspheric skin organoids based on spinning bioreactors. The organoid is featured by a core‐shell structure. Specifically, it consists of an inner core composed of dermal cells and collagen, and an outer shell comprising epidermal cells with full differentiation potential to form mature barrier, closely resembling the architecture of the human skin. As an in vitro skin model, microspheric skin organoids are suitable for various applications, including toxicity testing, and the screening for mediators in targeted signaling pathways such as Wnt/beta‐catenin pathway. Thus, our study promises the manufacture of microspheric skin organoids at a low cost, facilitating high‐throughput drug screening in the future.

## 5 Experimental Section

### Keratinocyte and Fibroblast Culture

HaCaT cells, human foreskin primary epidermal stem cells (Epi‐SCs), and human dermal fibroblasts (HDFs) were purchased from iCell Bioscience Co., Ltd (Shanghai). Both HaCaT and HDFs were cultured in DMEM (Corning) supplemented with 10% fetal bovine serum (FBS, Excell). Epi‐SCs were cultured in a growth medium (containing DMEM and F12 in a ratio of 1:3, 5% FBS, and 0.6 mm Ca^2^^+^). Cells were subcultured after trypsinization (0.05% trypsin and 0.02% EDTA, Thermo Fisher Scientific). For cryopreservation, cells were suspended in CELLBANKER2 (Amsbio, 11891) and frozen in −80 °C freezer for 24 h, and then in liquid nitrogen for the long‐term storage.

### Fabrication of Microspheric Skin Organoids

0.3 million HDFs were suspended in 600 µl type I collagen hydrogel (Corning, 354236, pH 7.0). 4 µl HDF/collagen hydrogel per well was added into 3M Novec 7100 fluid (methoxy‐nonafluorobutane) in Teflon‐96‐well plate. The HDF suspension was dripped into the wells to form HDF‐spheres (one drop per well). After incubation at 37 °C for 15 min, 200 µl DMEM (Corning, 10‐014‐CV) was supplemented to the wells, and HDF spheres were collected into aqueous phase. HDF‐spheres were rinsed with phosphate‐buffered saline (PBS, Corning) and incubated in non‐treated dishes in DMEM supplemented with 10% FBS. After incubation for 24 h, HDF‐spheres were transferred to spinner flasks (Integra Biosciences) and incubated with HaCaT cells in DMEM supplemented with10% FBS with a stirring speed of 20 rpm. After co‐culturing for 7 days, microspheric skin organoids formed with compartmented core‐shell structure. To achieve terminal differentiation of the epidermis, organoids were sequentially cultured in proliferation medium (containing DMEM and F12 in 1:3, 5% FBS, and 0.6 mm Ca^2^^+^) for 3 days, and subsequently in differentiation medium (containing DMEM and F12 in a ratio of 1:3, 5% FBS, 3.5 mm Ca^2^^+^, 5 µg ml^−1^ insulin, 0.5 µg ml^−1^ hydrocortisone, and 10 ng ml^−1^ epidermal growth factor (EGF)) according to previous studies.^[^ ^42^ ^]^

### Frozen Section

Samples were fixed in 4% paraformaldehyde (PFA, Sigma–Aldrich) at 4°C overnight, washed twice with PBS, and dehydrated in 10%, 20%, and 30% sucrose solutions at room temperature. Samples were then immersed in OCT Compound (SAKURA, 4583) and frozen in −80°C freezer. 8 µm‐thick sections were cut and stored at −20°C for the subsequent analysis.

### Immunofluorescence

Frozen sections were treated with 0.25% TritonX‐100 at room temperature for 30 min, blocked with 3% bovine serum albumin (BSA, Sigma–Aldrich)/PBS at room temperature for 1 h, incubated with primary antibodies at 37 °C for 2 h, and finally detected by fluorescence‐conjugated secondary antibodies at 37 °C in the dark for 30–60 min. Samples were imaged using a confocal microscope (Zeiss LSM‐780, German).

### Antibodies

Primary antibodies: rat anti‐Ki67 antibody (Invitrogen, PA5‐114437); rabbit anti‐caspase 3 (Genetex, GTX110543); mouse anti‐E cadherin antibody (Invitrogen, 13–1700); rat anti‐pan cytokeratin antibody; rabbit anti‐keratin 5 antibody (Abcam, ab52635); mouse anti‐ keratin 10 antibody (Invitrogen, MA1‐06319); mouse anti‐filaggrin antibody (santa cruz, sc‐66192); Secondary antibodies: goat anti‐rat 555 antibody (CST, 4417); goat anti‐rat 488 antibody (CST, 4416); goat anti‐rabbit 555 antibody (CST, 4413); goat anti‐mouse 555 antibody (CST, 4409); goat anti‐mouse 488 antibody (CST, 4408).

### Dual‐Luciferase Reporter System

For package of dual‐luciferase reporters into lentiviruses, three plasmids pCMV dR8.91, pMD2.G and target plasmids containing TCF/LEF: Fluc and hCMV‐IE1: Rluc were co‐transfected into 293T cells with polyethylenimine (PEI, Sigma–Aldrich), and after incubation1 for 2 h, the medium was changed to regular growth medium (DMEM supplemented with 10% FBS). HaCaT cells or HDFs were infected with lentiviruses containing the dual‐luciferase reporter system with 3 µg ml^−1^ puromycin (Sigma–Aldrich) for 48 h, and verified by examining their response to Wnt agonists and D‐luciferin (for Rluc).

### Lucifer Yellow Permeability Assay

The organoids were incubated with Lucifer Yellow (Sigma–Aldrich, L0259) at room temperature for 2 h. After washing with PBS, the organoids were embedded in the optimal cutting temperature compound and cryosectioned. Organoid sections were then examined under a confocal microscope (Zeiss LSM‐780, German). Cell nuclei were stained with DAPI.

### Organoids for Drug Testing

On the third day of organoid culture, the culture medium was carefully aspirated and discarded using a multichannel pipette, followed by the addition of serum‐free basal medium. 6 h later, the medium was replaced by fresh medium containing drugs and incubated for 24–48 h. Triplicate wells were used for each test. In testing of the effect of growth factors (EGF and bFGF), skin organoids were collected after treatment with different concentrations of the growth factors and embedded in the optimal cutting temperature compound. Cryosections in 250 µm thickness were subjected to immunofluorescence analysis. In luciferase reporter assay, after treatment with drugs, organoids were washed with PBS and lysed in a lysis buffer (YEASEN, 11402ES80) on ice. The lysate was then transferred to black 96‐well plates, and substrates of F‐Luc and R‐Luc were sequentially added. Illumination intensity was measured separately using a microplate reader. After normalization, the relative expression level of F‐Luc in each skin organoid was determined.

### Statistical Analysis

Values were presented as means ± SEM (*n* ≥ 3). Statistical significance was determined by Student's *t*‐test between two groups or One‐way ANOVA for analyses among multiple groups, and probability value (P) < 0.05 was regarded significant. The relevant graphs and analyses were completed using GraphPad Prism 8.0.1.

## Conflict of Interest

The authors declare no conflict of interest.

## Author Contributions

J.X. and Q.Y. contributed equally to this work. J.X. and Y.W. performed conceptualization. J.X. performed investigation. J.X., Y.Z., K.Z., H.G., and Q.Y. performed methodology. J.X. and Q.Y. performed formal analysis. J.X. and Q.Y. performed project administration. J.X. performed validation. Y.W. performed resources. J.X. performed and wrote the original draft. Y.W. performed funding acquisition. Y.W. performed wrote the original draft and edited. Y.W. performed supervision. All authors reviewed the manuscript.

## Supporting information



Supporting Information

## Data Availability

The data that support the findings of this study are available from the corresponding author upon reasonable request.
