# Supplementary material for: The Bioengineering of Microspheric Skin Organoids and Their Application in Drug Screening
Source: Adv Sci (Weinh). 2025 May 8;12(22):2416863. doi: 10.1002/advs.202416863 (PMC12165074; doi:10.1002/advs.202416863)

Supporting Information

The Bioengineering of Microspheric Skin Organoids and Their Application in Drug Screening

Jundong Xie, Qingyang Yang, Yanan Zhang, Ke Zheng Hongya Geng, Yaojiong Wu*

**Figure S1. Optimization of microspheric skin organoids.** (A). Contribution of HDFs to the size of HDF-spheres. Different numbers of HDFs (1000, 2000 and 4000 cells) were used to form HDF-spheres that were photographed at different time points during in vitro culture. (B). Effect of stirring speed in the cell spinner on adhesion of HaCaT cells. HDF-spheres (mCherry) and HaCaT cells (GFP) were co-cultured in the cell spinner at stirring speed of 20, 40, and 60 rpm. After 12 hours of incubation, HaCaT-HDF-spheres were photographed using fluorescent microscopy. (C). Effect of the ratio of keratinocytes to HDFs on the formation of epidermal layer. HaCaT cells (GFP) were co-cultured with HDFs at the ratio of 2:1, 5:1, 10:1 and 20:1. (D). Quantification of the size of HDF-spheres formed from different cell numbers. For each time point, ≥3 microspheres were analyzed. All values are presented as mean ± SEM. Error bars are not shown due to the similar size of HDF-spheres within each group. (E). Quantification of HaCaT coverage on the surface of HDF-spheres. All values are presented as mean ± SEM (n≥3). Statistical significance: one-way ANOVA (**P < 0.01). Scale bars: 100 μm.


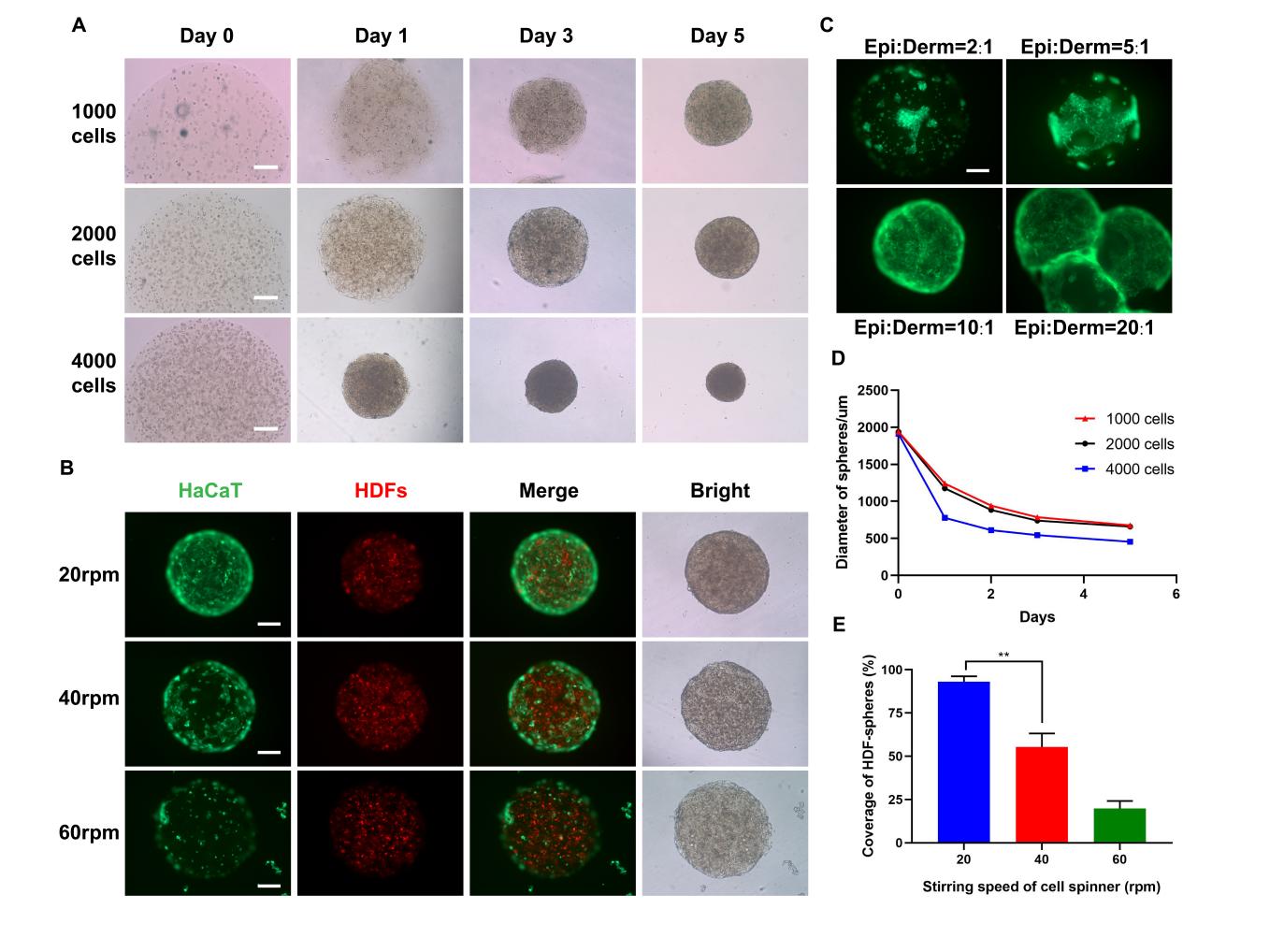


**Figure S2. Examination of the dual-luciferase reporter system for Wnt/beta-catenin signaling pathway.** (A). Examination of hCMV-IE1: Rluc domain. (B). Examination of TCF/LEF: Fluc domain after the treatment of 200ng/mL Wnt3a.


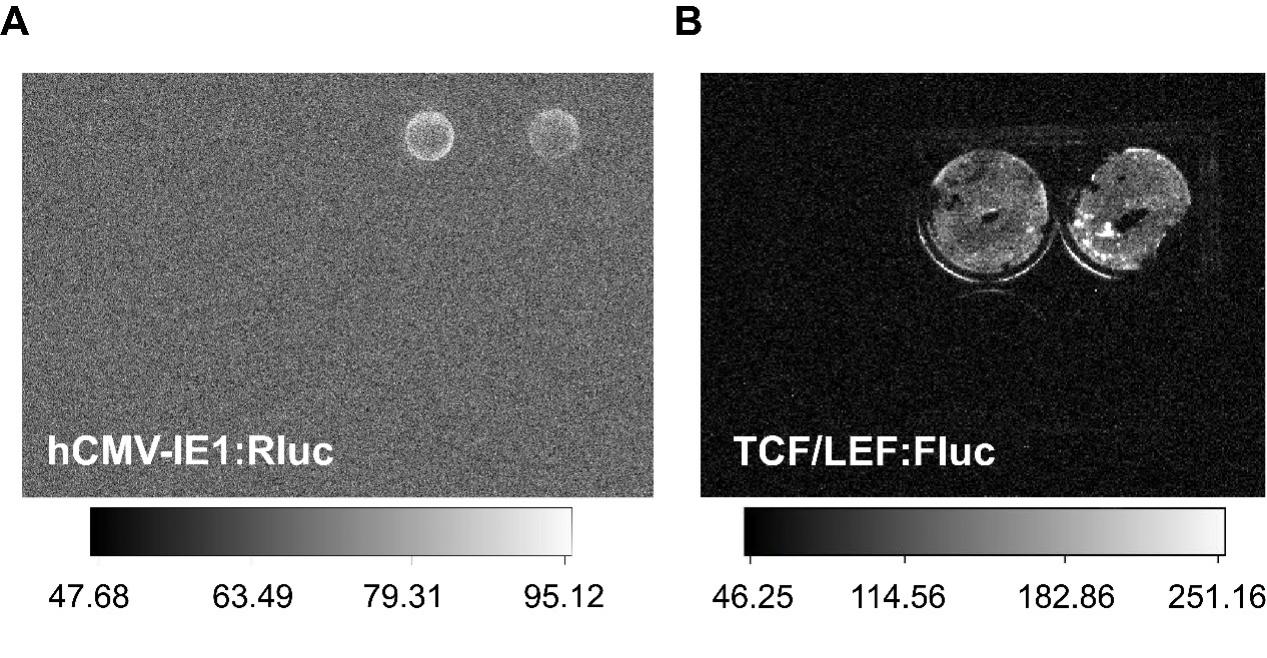


**Figure S3. The effect of Wnt agonist and growth factors on microspheric skin organoids comprising primary keratinocytes.** (A-B). Representative confocal images of the skin organoids cultured in growth medium (A) or differentiation medium (B) 48 h after drug supplementation. Organoids were treated with 0.1% DMSO (control), 20 ng/mL EGF, and 20 ng/mL bFGF, respectively. Keratinocytes were stained positive for E-cadherin (E-cad), nuclei were stained blue by DAPI, and proliferating cells were marked by Ki67. (C-D). Analysis of organoids treated with 0.1% DMSO (control), 5 μM CHIR-99021, 10 μM CHIR-99021, 20 ng/mL EGF, 40 ng/mL EGF, 20 ng/mL bFGF, and 40 ng/mL bFGF, respectively. The percentages of Ki67^+^ proliferating cells in the (C) epidermis or (D) dermis of the organoids were counted. Groups that showed significant differences between growth medium and differentiation medium were labeled by red stars. All values are presented as mean ± SEM (n ≥3), *P < 0.05. Scale bars: 100 μm.


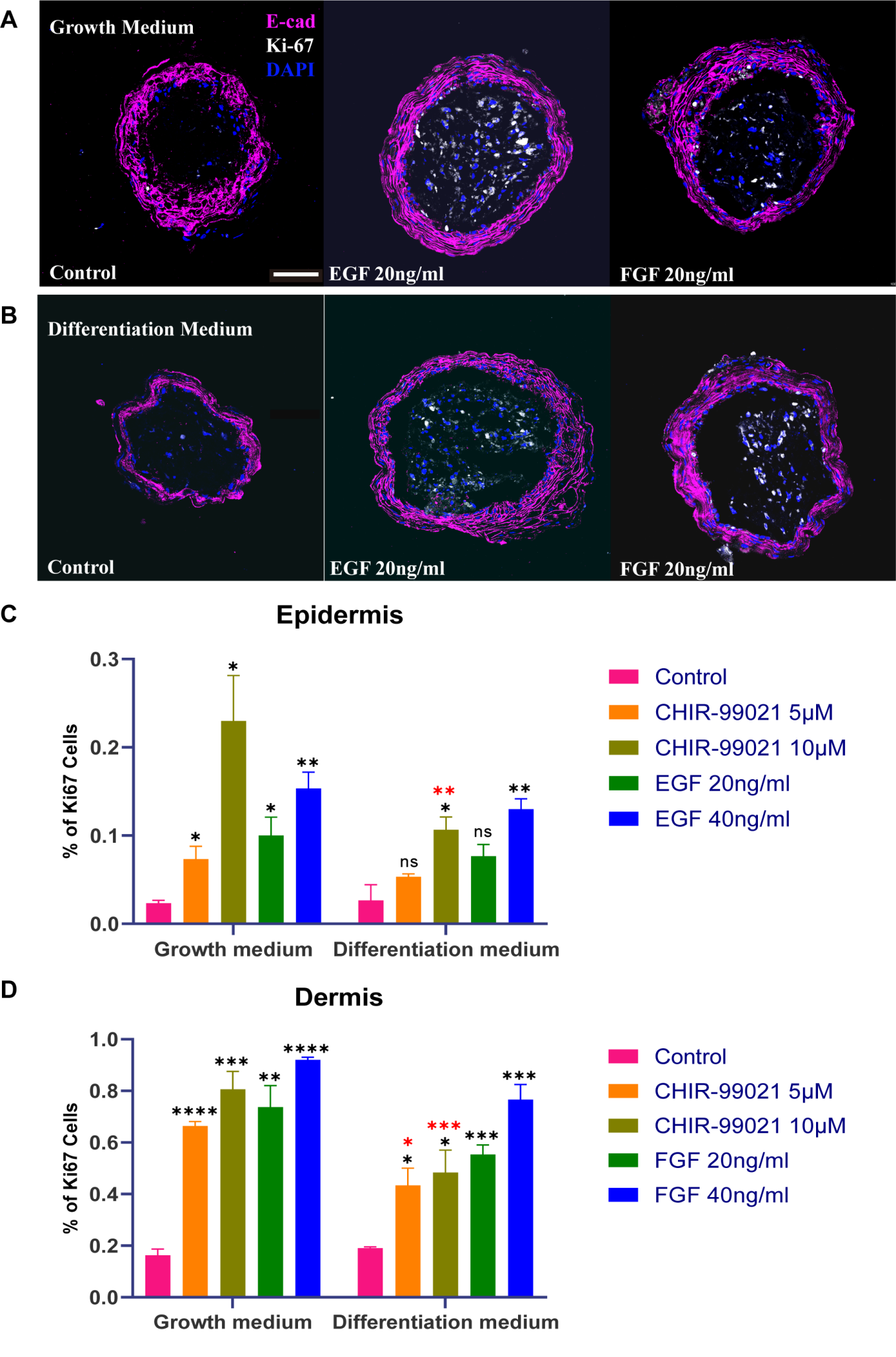

Supplement: Supplementary file 1 — Supporting Information [file ADVS-12-2416863-s001.docx]
